# Development of Leather-like Materials from Enzymatically Treated Green Kiwi Peel and Valorization of By-Products for Microbial Bioprocesses

**DOI:** 10.1021/acssuschemeng.5c04972

**Published:** 2025-09-18

**Authors:** Sara Mecca, Stefania Digiovanni, Riccardo Milanesi, Chiara Frigerio, Marco Mangiagalli, Giulia Tarricone, Matteo Boventi, Simone Bordignon, Michela Clerici, Marina Lotti, Roberto Simonutti, Luca Beverina, Paola Branduardi, Michele Mauri, Valeria Mapelli

**Affiliations:** † Department of Materials Science, 189823 University of Milano-Bicocca, Via Roberto Cozzi 55, Milano 20125, Italy; ‡ Department of Biotechnology and Biosciences, 9305 University of Milano-Bicocca, Piazza della Scienza 2, Milano 20126, Italy; § Department of Chemistry, University of Torino, Via P. Giuria, 7, Torino 10125, Italy

**Keywords:** biobased economy, waste valorization, leather-like materials, green kiwi peel, biomass treatment, yeasts

## Abstract

The management and possible valorization of agro-food waste is a major issue in the pursuit of a zero-waste economy. Green kiwi peel (GKP), the primary byproduct of kiwi fruit consumption, offers an attractive source of raw material for the development of biobased polymer films due to its availability and composition, which includes cellulose, hemicellulose, and pectin. In this study, we aimed to entirely valorize GKP by combining material functionalization and biomanufacturing approaches. Starting from mechanically ground GKP, either citric acid or two commercial enzyme preparations were employed to treat the biomass and obtain biobased films. Remarkably, the enzymatic treatment selectively consumes some of the component biopolymers, modifying their ratio. As a result, the mechanical properties of the GKP-derived films can be tuned depending on treatment conditions, offering the possibility of matching different requirements. We also show that the byproduct of biopolymer treatments, which is an acidic liquid fraction rich in glucose and fructose, can be used to formulate growth media for industrially relevant yeast cell factories, namely *Saccharomyces cerevisiae*, *Yarrowia lipolytica*, and *Rhodotorula toruloides*. Hence, the study presents a way to the full exploitation and valorization of the starting material. Overall, we propose an integrated approach with the aim of fully valorizing GKP, showing a versatile methodology that could be applied to other agro-food wastes to make them suitable for similar valorization pathways.

## Graphical abstract

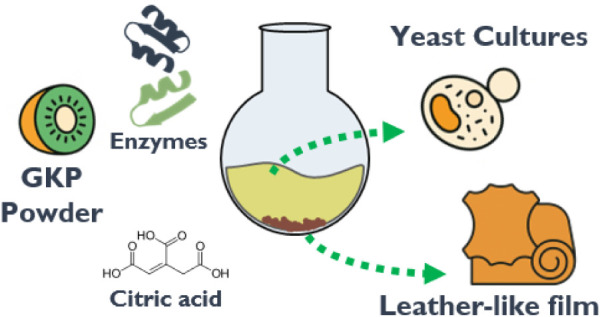

## Introduction

The agro-food industry generates large amounts of waste resulting from food production, processing, and consumption.^1^ The disposal and treatment of agro-food waste is a major global challenge with significant environmental, societal, and economic implications.^2,3^ Annually, the food industry produces approximately 490 million tons of fruit and vegetable waste, accounting for 38% of global food waste by mass.^2,3^ Improper management of this highly fermentable waste poses significant risks to human health and environmental integrity, potentially leading to issues such as eutrophication, soil acidification, and groundwater contamination.^4,5^ Even in the optimal scenario, where the edible part of fruits and vegetables is fully used for nutrition, with only minimal incidence of deterioration and discarding, the impact of the nonedible fraction on the overall waste management of cities is very sizable.^6,7^ Currently, anaerobic digestion and composting are the most common disposal methods for fruit and vegetable waste,^8,9^ aligning with regulations aimed at reducing landfill usage.^10^ While only a small fraction of fruit and vegetable waste can be used as animal feed, upcycling is emerging as a promising approach to reduce agro-food waste while creating value from discarded resources.^11,12^ The nonedible parts of fruits and vegetables, such as seeds, peels, husks, and pomace, are a good source of bioactive compounds,^13–15^ and raw materials for microbial or microalgae fermentation,^16–18^ and might have good potential for the development of sustainable biomaterials and bioplastics.^19–21^

Among fruits and vegetables, kiwi is an important source of antioxidant compounds, including vitamins A, C, and E, which are beneficial to human health.^22^ The pulp of this fruit is usually eaten as it is or processed to make fruit juices, sweets, and ice cream.^23,24^ World kiwi production reached an estimated 2.4 million tons in 2023, with 25 countries engaging in cultivation. China leads global production, followed by New Zealand, Italy, Greece, and Iran. Italy alone produced 477.5 kilotons of kiwi in 2023.^25,26^ The main byproduct of kiwi fruit consumption is its peel, which is still largely underexploited, as it is usually composted or subjected to anaerobic digestion.^23^ Recently, the green kiwi peel (GKP) has been preliminarily valorized through a chemical pretreatment with acetic acid and then used in the formulation of leather-like biomaterials with good mechanical properties.^27^

Biobased leather has emerged as an innovative and sustainable alternative to traditional animal and synthetic leather. These renewable materials are derived from diverse sources, including bacterial cellulose, mycelium, and plant and fruit waste.^28^ In the development of plant-based materials, the primary goal is to replace nonrenewable components of synthetic leather, such as polyvinyl chloride or polyurethane, with products derived from agricultural waste. Notable examples include materials made from grains, apple pomace (Vegea, Appleskin), ground cactus leaves (Desserto), and orange peel.^28,29^ The mechanical properties of the plant-based biomaterials depend mainly on the different proportions of the components of plant cell walls, such as cellulose (amorphous or microcrystalline), hemicellulose, lignin, and pectin.^19–21^ To enhance the cohesiveness of polysaccharide components and improve the mechanical properties of the resulting materials, vegetable waste typically requires physical and/or chemical pretreatments before biomaterial formulation and filming.^21,30^ Acid treatment has shown promising results in enhancing the film-forming capability of vegetable biomasses.^21,30,31^ While stronger inorganic acids are known for their high hydrolysis effect, the current trend favors the use of weak organic acids due to their lower corrosivity and reduced handling risks.^31,32^ However, it is important to note that even weak organic acids generate acidic fractions, which can pose disposal challenges.

In this work, we investigated the effects of different mild chemical or enzymatic treatments of GKP on the mechanical properties of leather-like biomaterials. The chemical treatment was performed using citric acid (CA), an odorless, bio-based, polyfunctional organic acid. The enzymatic treatment was performed using commercial multienzymatic preparations (enzyme cocktails), which are typically used for the degradation of plant biomass, especially its polysaccharide components. Both chemical and enzymatic treatments generate a secondary liquid stream, which poses a disposal challenge but, at the same time, provides organic-rich wastewater. The potential of these liquid fractions as carbon and nitrogen sources for the growth of industrially relevant yeast cell factories was evaluated. By valorizing both solid and liquid fractions of GKP, our method addresses waste management concerns while creating value-added products, thus promoting circular economy principles in the agro-food sector.

## Materials and Methods

### Materials

Green kiwis (*Actinidia deliciosa* variety) were purchased whole from a local grocery store (mixed country origin: Italy and New Zealand mainly). The enzymatic cocktails ViscozymeL and Cellic CTec2, citric acid, glucose, 2-(*N*-morpholino)­ethanesulfonic acid (MES), and CaCl_2_ were purchased from Merck (Merck, Darmstadt, Germany). Nitrogen quantification kits (l-Arginine/urea/ammonia assay kit and primary amino nitrogen assay kit) were purchased from Megazyme (Megazyme International, Bray, Ireland). The chemicals were employed as received without further purification. Polyvinylpyrrolidone (PVP, MW: 1,300,000) was purchased from Thermo Fisher Scientific (Thermo Fisher Scientific, Waltham, MA, USA), and polyglycerol (PG, pure vegetable polyglyceryl-3, Kosher grade, GMO-free) was purchased from Spiga Nord S.p.A. Reference compounds for HPLC analysis were purchased from Merck (Merck, Darmstadt, Germany). Yeast extract, tryptone, and agar were purchased from Thermo Fisher Diagnostics (Thermo Fisher Scientific, Waltham, MA, USA). (NH_4_)_2_SO_4_, NaOH and MgSO_4_ were purchased from Carlo Erba (Carlo Erba Reagents, Cornaredo, Italy).

### Mechanical Pretreatment and Characterization of GKP

The green kiwis were peeled manually with a knife, and the collected peels were washed with deionized water to remove impurities, dried in a ventilated dryer (90 °C for 16 h), coarsely chopped for 10 s with a blender to obtain cm-sized pieces, and then grinded in an ultracentrifugal mill (Retsch, ZM200) at 8500 rpm, adding them slowly to avoid material overheating. The resulting powder was sieved with a mechanical sieve shaker (Retsch, AS200 basic) using a 100 μm sieve. The GKP powder composition was qualitatively analyzed through solid-state nuclear magnetic resonance (SS-NMR) spectroscopy, while the morphology was studied with granulometry and scanning electron microscopy (SEM) analyses (described below). The surface charge was evaluated through zeta potential measurements.

### GKP Powder Treatments

#### Chemical Treatment of GKP Powder with CA

GKP powder was suspended in a 1 M aqueous solution of CA (10 mL/g dry biomass) in the presence or absence of CaCl_2_ (0.5 g/g dry biomass) for 24 h at 45 or 37 °C under magnetic stirring. The final dispersion was cooled to room temperature and centrifuged at 9000 rpm for 20 min. The liquid fractions (supernatant) were collected and stored at −20 °C, while the solid fractions (pellet) were directly employed for film preparation without any further treatment.

#### Enzymatic Treatments of GKP Powder

Two commercial enzyme cocktails, namely Viscozyme (VI) and Cellic CTec2 (CE), were employed. The enzymatic treatment of GKP was conducted in 15 mL plastic tubes (Euroclone S.p.A., Pero, Italy) with a working volume of 8 mL. Reactions were performed in distilled water at 37 or 50 °C for both enzyme cocktails, in the presence or absence of CaCl_2_ (50% *w/w* dry biomass). Tubes were shaken continuously at 500 rpm in a thermal shaker (Eppendorf, Hamburg, Germany). The enzyme loading was set at 0.5 mg of protein/g of dry biomass for both CE and VI, while the GKP powder content was 12.5% *w/v*. After the reaction, the final dispersion was cooled to room temperature and centrifuged at 9000 rpm for 20 min. The resulting solid (pellet) and liquid (supernatant) fractions were collected and pooled based on temperature and CaCl_2_ conditions.

### Characterization of the Solid Fractions Derived from GKP Treatments

#### Solid-State NMR Spectroscopy Analysis

The approximate composition of GKP powder was assessed by ^13^C solid-state NMR spectroscopy as previously described,^13^ employing a JEOL ECZR 600 NMR spectrometer (JEOL RESONANCE Inc., Japan) using magic-angle spinning (MAS) with a 3.2 mm rotor and a spinning rate of 10 kHz. The ^13^C cross-polarization (CP) MAS spectra were acquired with a relaxation delay of 3 s, a contact time of 2000 μs, and 4096 scans.

#### Granulometry Analysis

The particle size distribution was measured in deionized water at room temperature by using a Microtrac Sync 2R (Microtrac, Toulouse, France) equipped with a FlowSync wet dispersion unit. The reported distribution is the average of 3 runs of 30 s each.

#### Scanning Electron Microscopy (SEM) Analysis

The differences in morphology between untreated and treated GKP were evaluated by using field-emission SEM. The vacuum-dried powders were deposited onto carbon tape on an aluminum pin stub (top diameter 12.7 mm, standard pin) and coated with a gold–palladium mixture (Au–Pd 70–30%) under an argon atmosphere. Samples were analyzed by a Zeiss Gemini 500 instrument equipped with a QUANTAX EBSD (Electron Backscattered Diffraction) detector, and micrographs were obtained at an accelerating voltage of 3–5 kV.

#### Zeta Potential Analysis

Zeta potential analysis was performed using a Malvern Nano-S, whose data processing software is Zetasizer (Malvern). The refractive index (RI) of the dispersant, deionized water, was 1.330 with an absorption of 0.0 at the laser probe wavelength (633 nm). Analyses were performed on 1 mL samples at a concentration of about 50 μg/mL immediately after vortexing for 1 min to prevent aggregation, using a disposable folded capillary cell (DTS1070 type). Data were collected at 25 °C (equilibration time: 10 s). Three consecutive measurements were performed on each sample, with the number of runs ranging from 12 to 30 (automatically selected by the instrument). When data met the quality criteria (i.e., satisfying sample concentration, phase data plot, and counts) defined by the data analysis software, such measurements were considered reliable and were averaged to obtain the average zeta potential value and its standard deviation.

### Preparation and Characterization of GKP-Based Films

#### Film Preparation

Films were prepared as previously described.^27^ Briefly, the solid fraction obtained after GKP treatments was dispersed in deionized water (8 mL/g biomass) with polyvinylpyrrolidone (PVP, 20% w/w biomass) and polyglycerol (PG, 20% w/w biomass), which had been previously dissolved in deionized water overnight. The mixture was kept at 85 °C for 1 h under vigorous stirring. Then, the dispersions were poured through a food siever directly onto a silicon mat, and water was left to evaporate for 48 h at room temperature.

#### Mechanical Characterization of GKP Films

Mechanical properties of the films were measured by uniaxial tensile tests on a ZwickRoell 1445 dynamometer equipped with a RetroLine Test Control II unit using a 100 N load cell. Specimens were prepared by cutting films into 10 mm × 30 mm wide strips and were stretched at a rate of 1 mm/min. Each film underwent a minimum of three measurements on different sample parts, and the resultant values were averaged to derive a mean value. Young’s modulus (*E*), tensile strength (TS), and elongation at break values were determined from the stress–strain curves.

#### Water Contact Angle (WCA) Measurement

Contact angles were measured using a pocket goniometer, Fibro PGX plus, with 4 μL droplets of deionized water. Droplets were deposited and left on the surface for 2 min in the central section of the final film.

#### SEM Analysis of the GKP Films

The surface morphology of the films was analyzed by SEM. A piece of the film was placed as it was on carbon tape and then coated with a gold layer. The images were recorded as previously described for the powders.

### Growth of Yeast Strains on Treated GKP-Derived Liquid Fractions

GKP-derived liquid fractions were thawed at room temperature, diluted 1:4 with deionized water, pH-adjusted to 5 with NaOH, and then sterilized in an autoclave, followed by a filtration step. This dilution step was necessary to overcome sterilization challenges posed by the presence of residual CaCl_2_ from the GKP treatment (causing precipitates after heat sterilization) and by the high viscosity of the medium (preventing filtration). The resulting solutions are hereafter referred to as CA_GKP, VI_GKP, and CE_GKP media when obtained from CA, VI, and CE treatments, respectively. To test the effect of nitrogen on yeast cell growth, the GKP medium was supplemented with 5 g/L (NH_4_)_2_SO_4_ prior to sterilization.

#### Characterization of GKP-Based Growth Media

To characterize the monosaccharide content, the GKP media were appropriately diluted with ultrapure water (18 MΩ) and analyzed using an HPLC Agilent 1260 Infinity Quaternary LC (Agilent Technologies, US) equipped with an Agilent 1260 Infinity Refractive Index Detector (Agilent Technologies, US). Identification and quantification of monosaccharides were performed with a Rezex RPM-Monosaccharide Pb^2+^ Ion Exclusion 300 × 7.8 mm, 8 μm column (Phenomenex) thermostated at 40 °C, using ultrapure H_2_O (18 MΩ) as the mobile phase with a flow rate of 0.5 mL/min. Organic acids (i.e., D-galacturonic acid and CA) were identified and quantified using a Rezex ROA-Organic Acid H^+^ (8%) Ion Exclusion column 300 × 7.8 mm, 8 μm (Phenomenex) thermostated at 40 °C, using H_2_SO_4_ 0.005 N as the mobile phase at a flow rate of 0.5 mL/min. 10 μL samples were injected. Peaks were identified by comparison with reference standards dissolved in ultrapure H_2_O (18 MΩ) and quantified by using calibration curves prepared in a range between 0.625 and 20 g/L.

To quantify the available nitrogen sources (e.g., l-arginine, urea, ammonia, and primary nitrogen), 100 μL of GKP media was assayed using l-arginine/urea/ammonia (K-LARGE) and primary amino nitrogen assay (PANOPA) kits (Megazyme, IE). The absorbance at 340 nm was measured by using a Jasco V-770 UV/NIR spectrophotometer (JASCO Europe, Lecco, Italy).

#### Growth of Yeasts on GKP Media

Fresh cultures of *Saccharomyces cerevisiae* CEN.PK 113–7D, *Yarrowia lipolytica* W29 (CBS 7504), and *Rhodotorula toruloides* DSM 4444 were obtained with streaking glycerol stocks on YPD solid medium (2% (w/v) glucose, 2% (w/v) tryptone, 1% (w/v) yeast extract, and 2% (w/v) agar). Three independent colonies were inoculated in YPD liquid medium and grown until the midlate exponential phase.

For the spot tests, plates were prepared by mixing each GKP medium with a 40 g/L solution of agar in a 1:1 ratio. Cells in the midlate exponential phase were collected, washed with sterile deionized H_2_O, and diluted to the following concentrations: 10^6^ cells/mL, 10^4^ cells/mL, and 10^3^ cells/mL. 6 μL portions of each cell culture were spotted onto the agar plates. As a positive control, the same cell cultures were spotted on modified minimal medium agar plates (composition in Table S1). All the plates were then incubated for 2–3 days at 30 °C.

Growth was carried out in 250 mL shake flasks containing 50 mL of CA_GKP, VI_GKP, or CE_GKP media. Fresh yeast cultures were obtained as described previously, centrifuged, washed with sterile deionized H_2_O and resuspended in the growth medium to a starting concentration of 0.1 OD. Cell cultures were incubated at 30 °C for 48 h at 180 rpm. Cell growth was monitored by measuring OD_600_ with a Shimadzu UV1601 spectrophotometer (Shimadzu Italia, S.R.L.), while HPLC analyses were carried out to measure glucose and fructose concentrations over time as previously described.

## Results and Discussion

### Characterization of Untreated GKP Powder and Derived Films

The dried GKP was ground using an ultracentrifugal mill, obtaining a powder with a bimodal large particle size distribution (20 and 48 μm, Figure 1a), which also revealed an irregular shape, as confirmed by SEM images typical of plant-derived fibrous particle structures^33,34^ (Figure 1b), and was well distinguishable and coherent with wet granulometric analysis.

**Figure 1:**
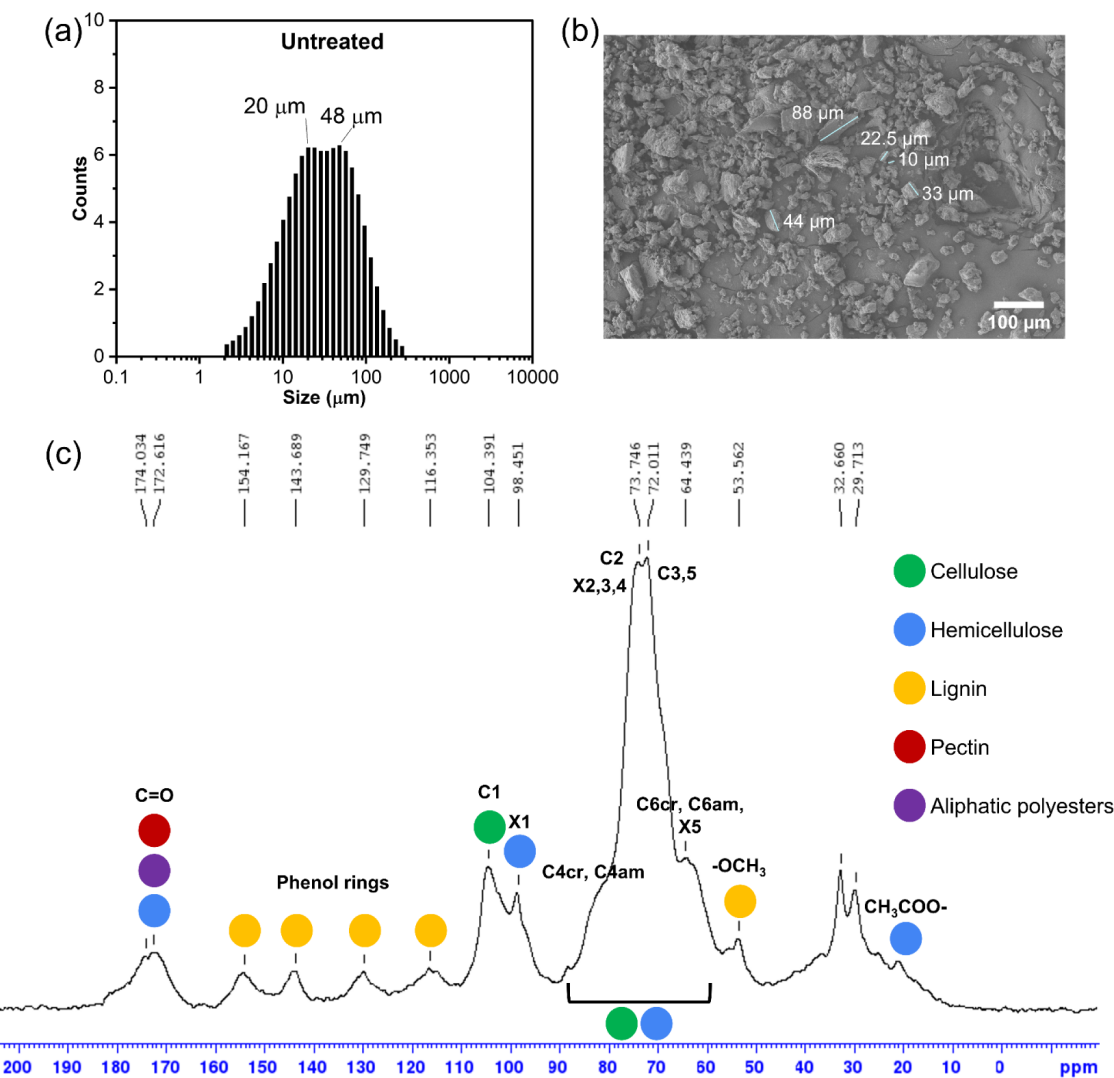
Characterization of the untreated GKP powder. (a) Bimodal particle size distribution derived from granulometric analysis in deionized water dispersion; (b) SEM image at 300× magnification with some particles highlighted by their longest dimension, and (c) CPMAS ^13^C NMR spectrum of the untreated GKP powder.

At the end of this process, ∼140 g of GKP powder (hereafter referred to as untreated GKP) was obtained from 750 g of nonedible waste parts (with high moisture content), which are derived from 3 kg of kiwi. The overall yield corresponds to around 4.6% GKP powder per kilogram of kiwi. The Solid-State NMR (SSNMR) spectrum of the sample (Figure 1c) revealed signals attributable to various components, including hemicellulose-type chains, cellulose, pectin, lignin, and some aliphatic polyesters.^20,35^ The spectrum was dominated by cellulose signals, with the most intense peaks observed in the chemical shift regions of 102–107 ppm, 80–92 ppm, and 60–67 ppm, corresponding to C1, C4, and C6 signals of glucose in cellulose, respectively. The C1 signal of hemicellulose appeared at ∼102 ppm, while its other signals overlapped with those of cellulose, displaying shoulders at chemical shifts lower than those of the cellulose C6 signal. Pectin signals were identified by peaks at ∼53 ppm (OCH3) and 172 ppm (carboxylic group). Lignin signals were observed in the 125–160 ppm region, while aliphatic polyester signals appeared at 30 ppm. It would be extremely valuable to retrieve a quantitative composition of the structural macromolecules identified, as studies on GKP composition have primarily focused on soluble bioactive compounds.^23^ However, the complexity and heterogeneity of the substrate result in a very complex SSNMR spectrum (Figure 1c). Hence, peak deconvolution is challenging, and the deriving percentage distribution of single macromolecules would result in a rough approximation. While such an analysis is beyond the scope of this study, it could be the subject for future development of a more accurate and quantitative method.

In the film obtained with untreated GKP powder (Figure 2a), no macroscopic fractures of the surface were observed, while microscopic analysis through SEM revealed the presence of natural aggregates embedded in the polymeric structure (Figure 2b), suggesting nonoptimal miscibility between the naturally derived part and the film-forming agents. The mechanical properties of the resulting biomaterial were characterized using several key parameters: Young’s modulus, tensile strength, and elongation at break. These properties provide insights into material ductility, resistance to elastic deformation, and the maximum stress it can withstand before failure when subjected to stretching or pulling forces. The stress–strain curves indicate mechanical rubber-like characteristics^36^ because of its high elongation at break and low Young’s modulus and tensile strength (Figure 2c,d). These results suggested that the starting material exhibits promising features. However, the introduction of a treatment step able to mildly modify, “activate”, the surface exposed structures, such as the organic acid or the enzymatic ones, is intended to improve some of the mechanical traits. This step favors cross-linking of different chains, enhances compatibility with low-polarity formulating materials, and, at the same time, creates the possibility to fine-tune final material properties as desired.^21,30,31^

**Figure 2:**
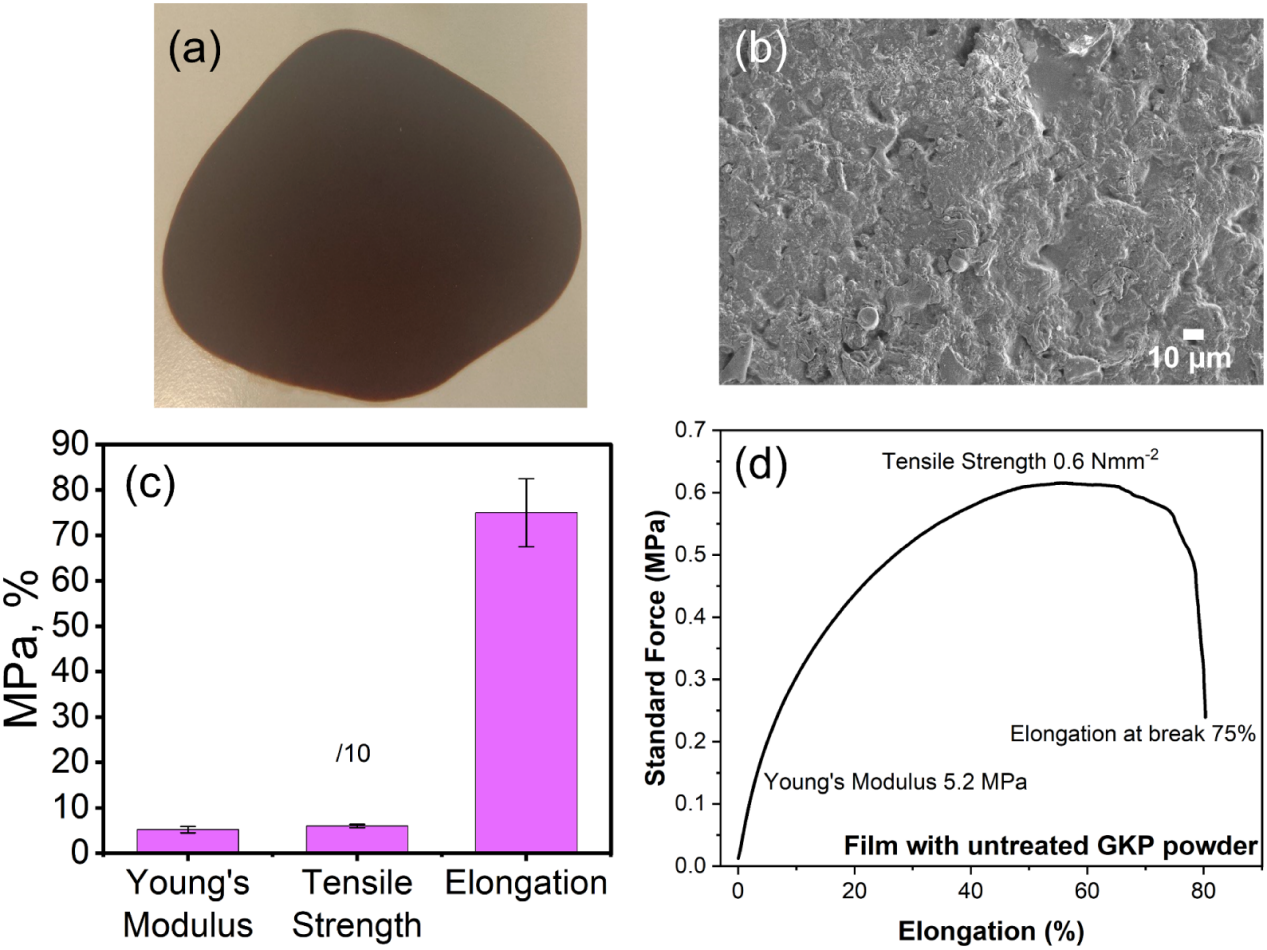
Structural and mechanical characterization of leather-like films from untreated GKP. (a) Photograph of the film obtained with untreated GKP powder, (b) SEM image at 1000× magnification, (c) main mechanical properties (averaged values from 3 samples of Young’s modulus, tensile strength, and elongation at break %); and (d) stress–strain curve of the film obtained with untreated GKP powder.

### Effects of Chemical and Enzymatic Treatments on GKP Powder

A recent patent application describes a mild chemical treatment with acetic acid that effectively modifies the structure of the GKP powder, resulting in the production of a film with mechanical properties closer to those of leather materials.^27^ In this study, we applied a different acid treatment using CA. This organic acid was chosen for being environmentally friendly, cost-effective, odorless, and biobased. Additionally, CA’s multifunctionality enables stronger interactions with the polar groups characteristic of natural material surfaces.^37^ To explore the array of possible modifications of the starting material more in depth, we assessed a novel approach based on the treatment of GKP with two commercial enzyme cocktails, that is VI and CE. These multienzymatic preparations, which are commonly used to treat lignocellulosic and plant biomasses, have different compositions. VI contains a variety of carbohydrate-active enzymes (CAZymes), including cellulases, hemicellulases, β-glucosidases, and pectinases.^38^ The exact composition of CE is proprietary, but notably, it lacks pectinases and includes lytic polysaccharide monooxygenase (LPMO),^39^ an enzyme absent in VI.^38^ Selecting these enzyme cocktails allowed us to investigate a broad spectrum of enzymatic modifications on GKP. For clarity, GKP after treatment with CA, VI, and CE are hereafter referred to as CA_GKP, VI_GKP, and CE_GKP, respectively.

The conditions used for the CA treatment are the same as those described in ref 27, and include the hydrolysis at 45 °C and the addition of CaCl_2_, which is critical for providing the film with the necessary mechanical properties.^40^ This treatment resulted in water-dispersed CA_GKP particles of a size comparable to that of the untreated GKP (Figures 1a and 3a) and with a slightly reduced content of pectin, lignin, and hemicellulose, as suggested by SSNMR (Figure 3g). SEM imaging highlighted the increased compactness of CA_GKP particles under water evaporation for sample preparation compared to the untreated ones (Figures 1b and 3d), likely due to the cross-linking of both CaCl_2_ and CA with the structural polysaccharides. Increased magnification showed needlelike structures (Figure S1), attributable to the formation of calcium citrate crystals.^41^ This hypothesis is supported by zeta potential analysis, which showed that the negative charge decreased from −24.8 ± 3.5 mV in untreated GKP to −12 ± 0.4 mV in CA_GKP.

**Figure 3:**
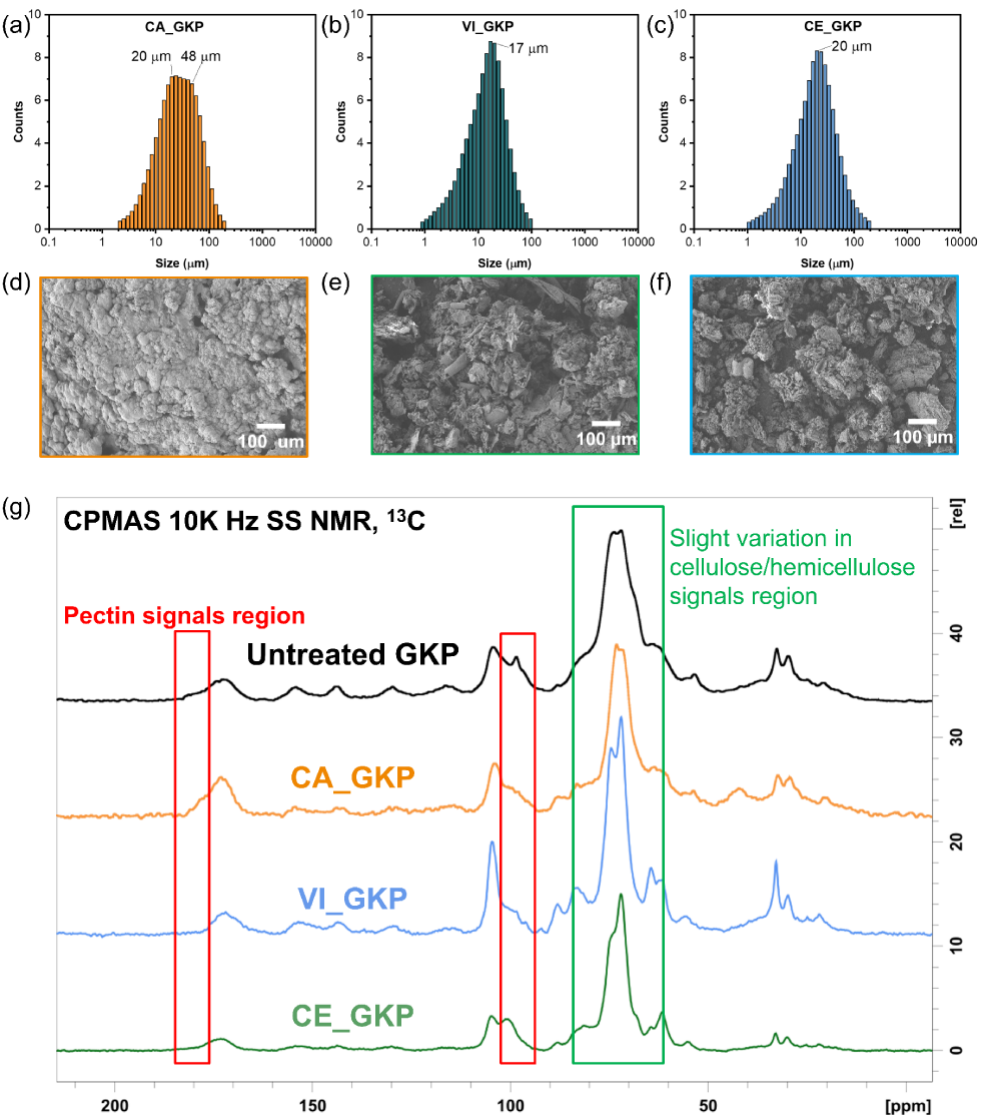
Characterization of the treated GKP powder. Granulometric analysis in water dispersion of CA_GKP (a), VI_GKP (b), and CE_GKP (c). SEM images at 300× magnification of CA_GKP (d), VI_GKP (e), and CE_GKP (f) particles. (g) CPMAS 13C-NMR spectra comparison, with regions that differ from the untreated GKP powder spectrum highlighted in colors corresponding to the different components (red for pectin, green for hemicellulose/cellulose). The GKP treatments reported in this figure were carried out with CA at 45 °C and VI and CE at 37 °C. All treatments were performed in the presence of CaCl_2_.

Enzymatic treatment was performed at 37 °C for 24 h at a low enzyme load (0.5 mg/g of dry GKP). These conditions, suboptimal compared to those specified by the manufacturer (i.e., 50 °C), were chosen to mildly hydrolyze the structural polymers present in the GKP powder, as partially maintaining polymeric structures could be critical for the final properties of the leather-like material. At the end of the treatment, both CE_GKP and VI_GKP particles had a monomodal particle size distribution, with an average size of 20 and 17 μm, respectively (Figure 3b,c). The measurement is performed by analyzing diluted water dispersions of the samples, allowing the different particles to be detected by the instrument camera. The charges measured by zeta potential were −10.3 ± 1.9 mV for CE_GKP and −18 ± 1.2 mV for VI_GKP, which were lower than the charges measured on untreated GKP. SEM analyses of both enzymatically treated powders, directly deposited on the analysis stub and then dried under laboratory atmosphere, showed the formation of agglomerates (Figure 3e,f), indicating increased cross-linking of the polysaccharide chains when excess water is removed, compared to untreated particles that remain separated (Figure 1a,b). The SSNMR spectra revealed slight variations in the relative intensities of the signals associated with cellulose and hemicellulose chains, indicating that both enzyme cocktails partially hydrolyzed these components of GKP (Figure 3g). The VI enzyme preparation was able to degrade pectin, as indicated by the decrease in the peak at 170 ppm; such activity was corroborated by the release of galacturonic acid, which is the main product of pectin hydrolysis, detected by HPLC analysis (data not shown).

### Characterization of GKP Films under Different Treatments

GKP films were formulated as described in the Materials and Methods section. Treatment with CA at 45 °C or enzymatic cocktails at 37 °C resulted in more homogeneous materials compared to films derived from untreated GKP (Figure 4a–c compared to Figure 2a). Young’s modulus of the CA_GKP film (8.0 ± 2.6 MPa) was found to be significantly lower than those obtained with VI_GKP (20.5 ± 0.8 MPa) and CE_GKP (24.0 ± 1.5 MPa) (Figure 4d–f), and more similar to that of the untreated GKP film (5.2 ± 0.7 MPa). On the other hand, the tensile strength is very similar regardless of the treatments (0.6 ± 0.1 MPa, 1.0 ± 0.1 MPa, and 1.0 ± 0.1 MPa, respectively), and also with respect to the untreated GKP film (0.6 ± 0.04 MPa). The CA_GKP (43.0 ± 3.0%) and VI_GKP (42.0 ± 3.0%) films exhibit similar elongation, which is 1.6 times higher than the value observed for the CE_GKP film (26.0 ± 4.0%) (Figure 4d–f). The microstructure of GKP films was investigated by SEM (Figure 4g–i), with only minor differences visible at the highest magnifications (Figure S2).

**Figure 4:**
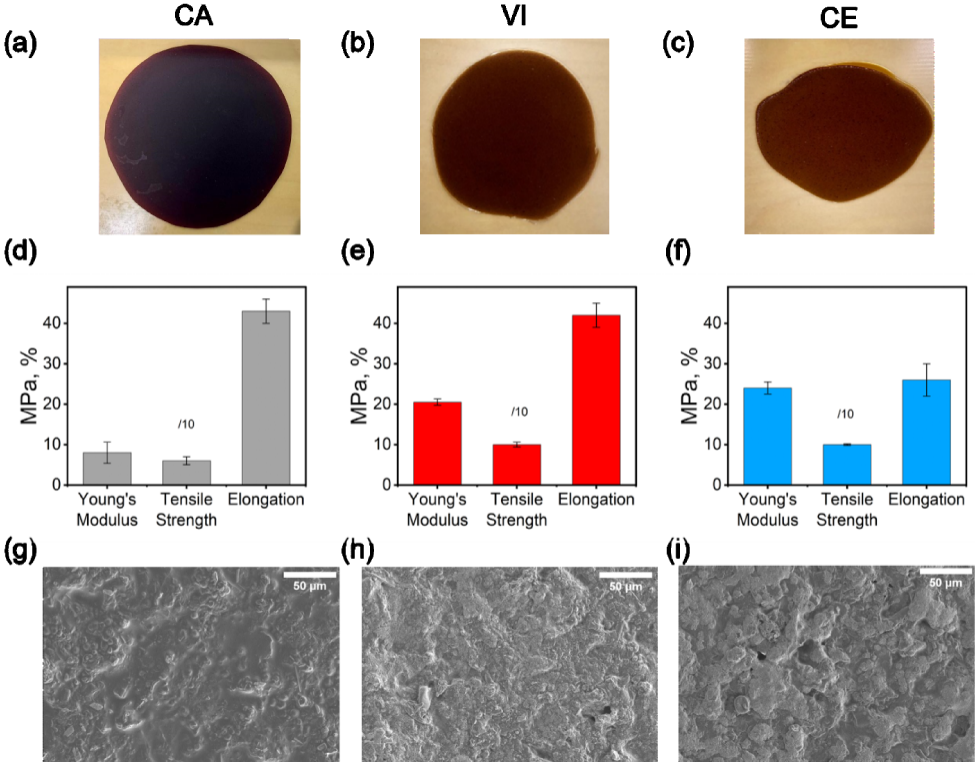
Properties of the GKP films. (a–c) Images of CA_GKP (a), VI_GKP (b), and CE_GKP (c) films. (d–f) Mechanical properties of CA_GKP (d), VI_GKP (e), and CE_GKP (f) films, with values listed in Table 1. (g–i) SEM images at 1000× magnification of CA_GKP (g), VI_GKP (h), and CE_GKP (i) films. The films reported in this figure were obtained by treating GKP with CA at 45 °C and VI and CE at 37 °C. All treatments were performed in the presence of CaCl_2_.

The WCA measurements indicate that the CA_GKP film is the most hydrophilic (68.3° at 0 min) among those obtained in this study upon acidic or enzymatic (VI 105.8° and CE 106° at 0 min) pretreatment (Table 1), probably related to the high content of CA retained in the treated particles (as also confirmed by characteristic peaks in the SSNMR spectra, Figure 3g). Both enzyme-treated films initially exhibit enhanced hydrophobicity with respect to the acid-treated one, as indicated by higher contact angle values. However, these values can still be improved with the addition of a proper hydrophobic coating layer,^42^ especially since the contact angle value decreases after 2 min (for VI from 105.8° to 62.0° and for CE from 106.0° to 62.2° in 2 min), suggesting that water penetration into the film structure still occurs over time, allowing the droplet to swell within the material (Table 1).

**Table 1:** Mechanical Properties Measured by Dynamometer Analysis and Water Contact Angles (WCA) of Films Obtained through Different GKP Pretreatments

Treatment	Temperature (°C)	Young modulus (MPa)^a^	Tensile strength (MPa)^a^	Elongation at break (%)^a^	θ°, 0 min, air^a^	θ°, 2 min, air^a^
Untreated		5.2 ± 0.7	0.6 ± 0.1	75.0 ± 7.5	97.6	46.3
CA	45	7.2 ± 0.3	0.3 ± 0.1	25.0 ± 2.0	72.0	37.0
CA + CaCl_2_	45	8.0 ± 2.6	0.6 ± 0.1	43.0 ± 3.0	98.9	65.4
CA + CaCl_2_	37	3.5 ± 0.7	0.6 ± 0.2	71.0 ± 22	68.3	30.9
VI	37	50.0 ± 6.0	2.4 ± 0.3	19.8 ± 5.5	113.3	71.5
VI + CaCl_2_	37	20.5 ± 0.8	1.0 ± 0.1	42.0 ± 3.0	105.8	62.0
CE	37	25.3 ± 4.0	1.2 ± 0.1	15.2 ± 2.3	101.5	73.3
CE + CaCl_2_	37	24.0 ± 1.5	1.0 ± 0.1	26.0 ± 4.0	106.0	62.2
VI + CaCl_2_	50	162.0 ± 15.0	5.7 ± 0.3	11.0 ± 2.0	28.3	Adsorbed
CE + CaCl_2_	50	64.5 ± 23.2	4.5 ± 0.7	15.0 ± 2.0	102.1	51.2

a Values are the mean of three independent measurements.

Overall, the assessment of the mechanical properties of the GKP films obtained after treatment with CA or the enzymatic cocktails revealed that, in comparison with the acetic acid treatment,^27^ the Young’s moduli were lower (between 8.0 and 24.0, compared to the AA treatment at 88 ± 7 MPa^27^), while the elongations at break were higher (between 23% and 43% with respect to the AA treatment 14% ± 7 ^27^). To understand the effect of other variable pretreatment parameters on the film performances, different temperatures and the presence/absence of CaCl_2_ were considered. The mechanical properties of the films obtained under different treatment conditions are summarized in Table 1. The presence of CaCl_2_ increases the elongation at break of GKP films regardless of the treatment (Figure S3 and Table 1). Since CaCl_2_ slightly affects the composition of the GKP powder (Figure S4), this mechanical feature is likely due to the ability of the salt to create elastic cross-linking points.

To determine the effect of temperature, the CA treatment was evaluated at 37 °C in direct comparison with enzymatic conditions. The CA_GKP film obtained at 37 °C displays mechanical properties (Young’s modulus: 3.5 ± 0.7 MPa, tensile strength: 0.6 ± 0.2 MPa, and elongation at break: 71.0 ± 22.0%) more similar to those obtained with untreated GKP (Young’s modulus: 5.2 ± 0.7 MPa, tensile strength: 0.6 ± 0.1 MPa, and elongation at break: 75.0 ± 7.5%) compared to those treated at 45 °C (Young’s modulus: 8.0 ± 2.6 MPa, tensile strength: 0.6 ± 0.1 MPa, and elongation at break: 43.0 ± 3.0%). Considering that acid hydrolysis is often more efficient at higher temperatures, these results might indicate that the degree of hydrolysis can tune the mechanical properties of the GKP film (Figure S5 and Table 1). Therefore, the effect of a higher temperature (50 °C) was also assessed for the enzymatic treatments. As expected, CE catalyzed higher degradation of cellulose and hemicellulose components at 50 °C compared to 37 °C (Figure S6a). In contrast, VI activity on cellulose and hemicellulose is less affected, with a surprisingly reduced degradation of pectin (Figure S6b). In both cases, the formation of shorter chains with a higher number of exposed −OH groups occurring at 50 °C resulted in increased hydrophilicity (28.3° for VI and 102.1° for CE at 0 min; Table 1) and stiffness (Young’s modulus: 162.0 ± 15.0 MPa for VI and 64.5 ± 23.2 MPa for CE, Figure S7 and Table 1) of the final material. The tensile strength of GKP films (5.7 ± 0.3 MPa for VI and 4.5 ± 0.7 MPa for CE) is higher compared to that of Muskin, a mycelium-derived leather (0.2 MPa), but lower than other plant-based leathers, which range from 5 to 20 MPa; however, the elongation at break (11.0 ± 2.0% for VI and 15.0 ± 2.0% for CE) is similar to the latter (around 50.0%).^28,43^

In conclusion, while all three treatments produce similar materials, the enzymatic cocktails offer greater tunability of the mechanical properties. The proposed treatments provide a mild and straightforward method to enhance the properties of films produced from waste GKP. An interesting further improvement to be considered can be in the direction of a hydrophobicity increase, especially for applications in which higher water resistance is essential. A similar treatment involving acetic acid and avocado peel and seeds as substrates produced biofilms with a Young’s modulus (38–460 MPa) and tensile strength (0.6–18.5 MPa), depending on avocado part and plasticizers.^44^ These values are higher compared to those of GKP-treated biofilms at 37 °C. By contrast, the elongation at break reported for avocado-based biofilms is dramatically lower (3.9–19.8%) compared to those of GKP-based biofilms obtained after the treatment at 37 °C (see Table 1). The mechanical properties of the enzymatically treated GKP films at 50 °C aligned well with the avocado-based materials, especially in terms of tensile strength and elongation at break. This evaluation suggests that these treatments can be performed on a wide variety of substrates and under many different combinations of conditions, tuning the mechanical properties of the resulting films.

### Growth of Yeasts in Media Containing the Liquid Waste Stream from GKP Treatments

The liquid fractions obtained after GKP treatments exhibit an acidic pH (pH ∼3.0) and contain the organic matter released during pretreatment. Disposal of these fractions can be challenging. In the framework of a zero-waste economy, the potential of these liquid fractions as a source of nutrients for yeasts was investigated. More in detail, we tested the growth of *S. cerevisiae*, one of the most intensively studied unicellular eukaryotes both as a model organism and as an industrial workhorse, and the nonconventional yeasts *Y. lipolytica* and *R. toruloides*, showing increasing potential in industrial applications. These yeasts are broadly used to produce relevant biochemicals and as hosts for the heterologous production of proteins.^45,46^

The liquid fractions were diluted (1:4) to reduce viscosity and CaCl_2_ concentration, adjusted to pH 5.0 with NaOH, and used as the culture media. The presence of sugars in CA_GKP, VI_GKP, and CE_GKP-derived media was quantified by HPLC analysis. The measured total sugar content was between 10 and 11 g/L, with comparable amounts of glucose and fructose in all tested media. Specifically, considering the quantification of glucose and fructose, the overall sugar content was 11.1 g/L for CA_GKP, 9.6 g/L for VI_GKP, and 10.8 g/L for CE_GKP (Table 2), regardless of the standard deviations.

**Table 2:** Composition of GKP-Derived Media

	CA_GKP	VI_GKP	CE_GKP
Glucose (g/L)	4.6 ± 0.5	4.6 ± 0.4	5.7 ± 0.9
Fructose (g/L)	6.5 ± 0.9	5.0 ± 0.8	5.1 ± 0.2
Primary amino nitrogen (mg/L)	0.2 ± 0.1	6.7 ± 0.4	4.0 ± 0.3
Ammonia (mg/L)	n.d.	1.2 ± 0.3	1.0 ± 0.1
Urea (mg/L)	n.d.	n.d.	n.d.
l-Arginine (mg/L)	n.d.	n.d.	n.d.

Besides carbon and energy sources, yeast cells require either organic or inorganic reduced nitrogen sources to sustain their growth. Among the different available nitrogen species, ammonia salts and certain amino acids (such as glutamine, leucine and branched-chain amino acids) positively promote cell growth through the activation of protein synthesis.^47^ Considering this, the availability of urea, ammonia, arginine, and primary amino nitrogen was measured for all GKP-derived media (Table 2). The CA_GKP-derived medium contained below 1 mg/L of primary amino nitrogen, while ammonia, urea, or l-arginine were not detected. In the VI_GKP- and CE_GKP-derived media, the amount of primary amino nitrogen and ammonia salts was similar and 1 order of magnitude higher than in CA_GKP. These differences could derive from ammonia salts used during the downstream process of enzyme cocktails,^48^ while higher primary amino nitrogen is due to the presence of enzymes. Nevertheless, ammonia salts available in GKP-derived media are 3 orders of magnitude lower than the standard concentration for minimal media used for yeast growth.^49^

The ability of the selected yeast cell factories to grow on GKP-derived media with or without supplementation with 5 g/L (NH_4_)_2_SO_4_ was evaluated by a spot assay on solid media. In the absence of (NH_4_)_2_SO_4_, all yeast strains were able to grow on the VI_GKP- and CE_GKP-derived media, however, they formed colonies of a smaller size than those formed on the minimal medium, used as a positive control (Figure 4a). On the CA_GKP-derived medium, only *Y. lipolytica* formed colonies that were similar in size to those observed on the control medium. This is likely due to the ability of *Y. lipolytica* to tolerate high CA concentrations.^50^ Conversely, in the presence of (NH_4_)_2_SO_4_, all yeast strains formed colonies comparable to those obtained in the control medium. This finding suggests that the main impact of the GKP-derived media on yeast cell growth is possibly related to the available levels of nitrogen (Figure 5a).

**Figure 5:**
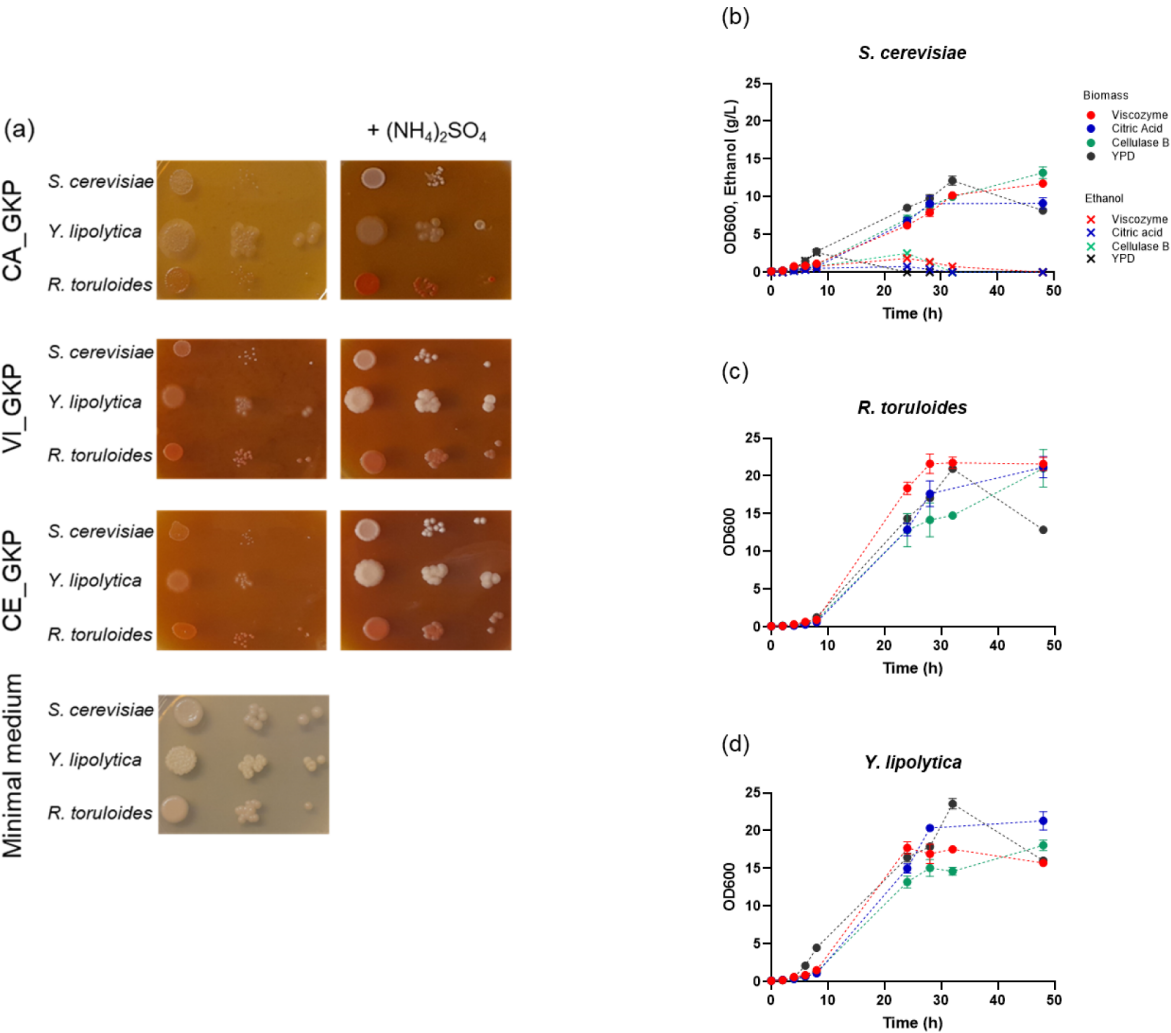
Growth of industrially relevant yeast cell factories on GKP-derived media. (a) Exponentially growing cell cultures were serially diluted (1:10), and each dilution was spotted onto GKP-derived medium plates with or without 5 g/L ammonium sulfate. A modified minimal medium containing 10 g/L glucose was used as a control. Growth curves of *S. cerevisiae* (b), *Y. lipolytica* (c), and *R. toruloides* (d) in CA_GKP (blue), VI_GKP (red), and CE_GKP (green) liquid media supplemented with 5 g/L ammonium sulfate. Biomass (OD_600_) and ethanol concentration (g/L) are indicated by full circles and crosses, respectively. Values are the mean ± standard deviation of three independent experiments.

To confirm the data obtained by the spot test on solid media, shake flask cultures were performed in GKP-derived media supplemented with 5 g/L of (NH_4_)_2_SO_4_. The growth of each tested strain in GKP-derived media was comparable to that in rich YPD media (Figure 5b-d), indicating the availability of essential trace elements and vitamins in GKP-derived media and no inhibitory effects from compounds such as polyphenols and galacturonic acid released during the treatments. Among the tested strains, the lowest biomass concentration was reached by *S. cerevisiae* due to the so-called “Crabtree effect”, whereby some of the available sugars are fermented to ethanol, resulting in the reduction of biomass yield.^51^ Overall, these observations demonstrate the applicability of liquid waste from the different GKP treatments for the cultivation of industrially relevant yeasts.

## Conclusions

The valorization of nonedible biomass, such as peels, seeds, and petals of flowers, is crucial for reducing disposal-related issues while creating new value chains. Overall, our data indicate that enzymatically treated GKP can be used in the formulation of leather-like materials with mechanical properties and appearance competitive with those previously described. Interestingly, enzymatic treatments can effectively replace organic acids in GKP processing. The mechanical and surface characteristics of the obtained materials show that enzymatic treatments can serve as a viable alternative to conventional chemical processes and offer the possibility to tune the performance of the final materials by varying the treatment conditions. The correlation between treatment conditions and final material properties, without precise specificity for GKP components, suggests the potential for adaptation of these treatments to other biomasses that can be used for similar applications.

In the context of full valorization of waste GKP, this research also highlights the potential of the liquid fraction derived from GKP treatment. Both acid-treated and enzyme-treated liquid byproducts can serve as growth media for industrially relevant yeasts, further improving the overall resource efficiency of the process. This integrated biorefinery approach exemplifies a comprehensive strategy for agro-food waste utilization, contributing to sustainable resource use and environmental stewardship.

## Supplementary Material



## Notes

Additional experimental details, materials, including ^13^C NMR spectra, stress–strain curve of a film, photographs of GKP-derived materials, images captured via SEM, and medium composition (PDF)
