# Supplementary material for: Development of Leather-like Materials from Enzymatically Treated Green Kiwi Peel and Valorization of By-Products for Microbial Bioprocesses
Source: ACS Sustain Chem Eng. 2025 Sep 18;13(38):15924–34. doi: 10.1021/acssuschemeng.5c04972 (PMC12486432; doi:10.1021/acssuschemeng.5c04972)
Supplement: Supplementary file 1 [file sc5c04972_si_001.pdf]

Supporting Information for

Development of leather-like materials from enzymatically treated green kiwi peel and valorization of by-products for microbial bioprocesses

Sara Mecca[a†], Stefania Digiovanni[b†], Riccardo Milanesi[b†], Chiara Frigerio[b†], Marco Mangiagalli[b], Giulia Tarricone[a], Matteo Boventi[a], Simone Bordignon[c], Michela Clerici[b], Marina Lotti[b], Roberto Simonutti[a], Luca Beverina[a], Paola Branduardi[b], Michele Mauri[a], Valeria Mapelli[b]**

[a] Department of Materials Science, University of Milano-Bicocca, Via Roberto Cozzi 55, 20125 Milano, Italy

[b] Department of Biotechnology and Biosciences, University of Milano-Bicocca, Piazza della Scienza 2, 20126 Milano, Italy

[c] Department of Chemistry, University of Torino, Via P. Giuria, 7, 10125 Torino, Italy

Page S2…………………………………………..…………………………..Table S1.

Page S3………………………………………….………………………Figure S1

Page S4………………..……………………………………………….…Figure S2

Page S5 ………………………………………………………… …..Figure S3, Figure S4

Page S6……………………………………………………………….….Figure S5

Page S7……………………………………………………………………..Figure S6

Page S8…………………………………………………………...……Figure S7

Number of pages: 8

Number of figures: 7

Number of tables: 1

Table S1. Composition of minimal medium and element trace solution.

Component	Concentration
Minimal medium	
Glucose	10 g/L
$(NH_4)_2SO_4$	5 g/L
KH_2PO_4	3 g/L
$MgSO_4 \cdot 7H_2O$	0.5 g/L
D-biotin	0.1 mg/L
calcium D-pantothenate	2 mg/L
nicotinic acid	2 mg/L
myo-inositol	50 mg/L
thiamine hydrochloride	2 mg/L
pyridoxal hydrochloride	2 mg/L
para-aminobenzoic acid	0.4 mg/L
MES	100 mM
Metal trace solution	
EDTA	30 mg/L
$ZnSO_4 \cdot 7H_2O$	9 mg/L
$CoCl_2 \cdot 6H_2O$	0.6 mg/L
$MnCl_2 \cdot 4H_2O$	2 mg/L
$CuSO_4 \cdot 5H_2O$	0.6 mg/L
$CaCl_2 \cdot 2H_2O$	9 mg/L
$FeSO_4 \cdot 7H_2O$	6 mg/L
$Na_2MoO_4 \cdot 2H_2O$	0.8 mg/L
H_3BO_3	2 mg/L
KI	0.2 mg/L

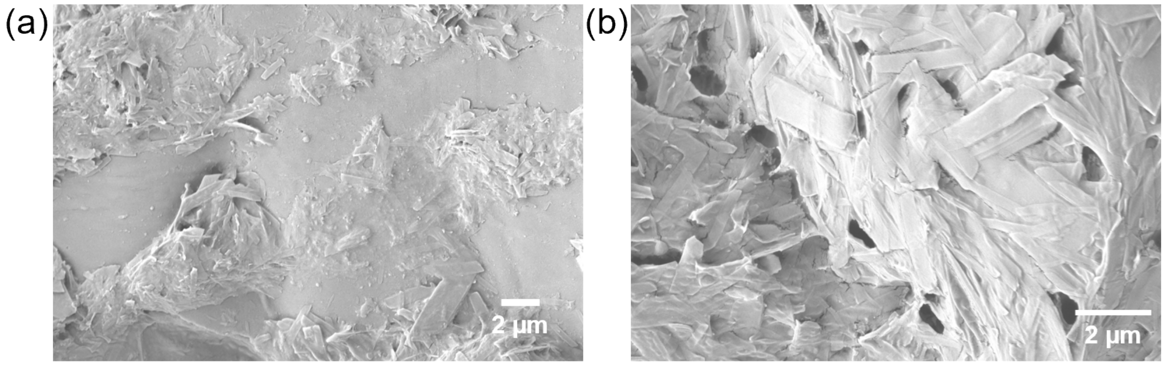

Figure S1. SEM images with (a) 10.000 X and (b) 20.000 X magnification of CA_GKP film, showing the presence of superficial needle-like structures that can be associated with the formation of calcium citrate crystals (Rimsueb et al., 2020).

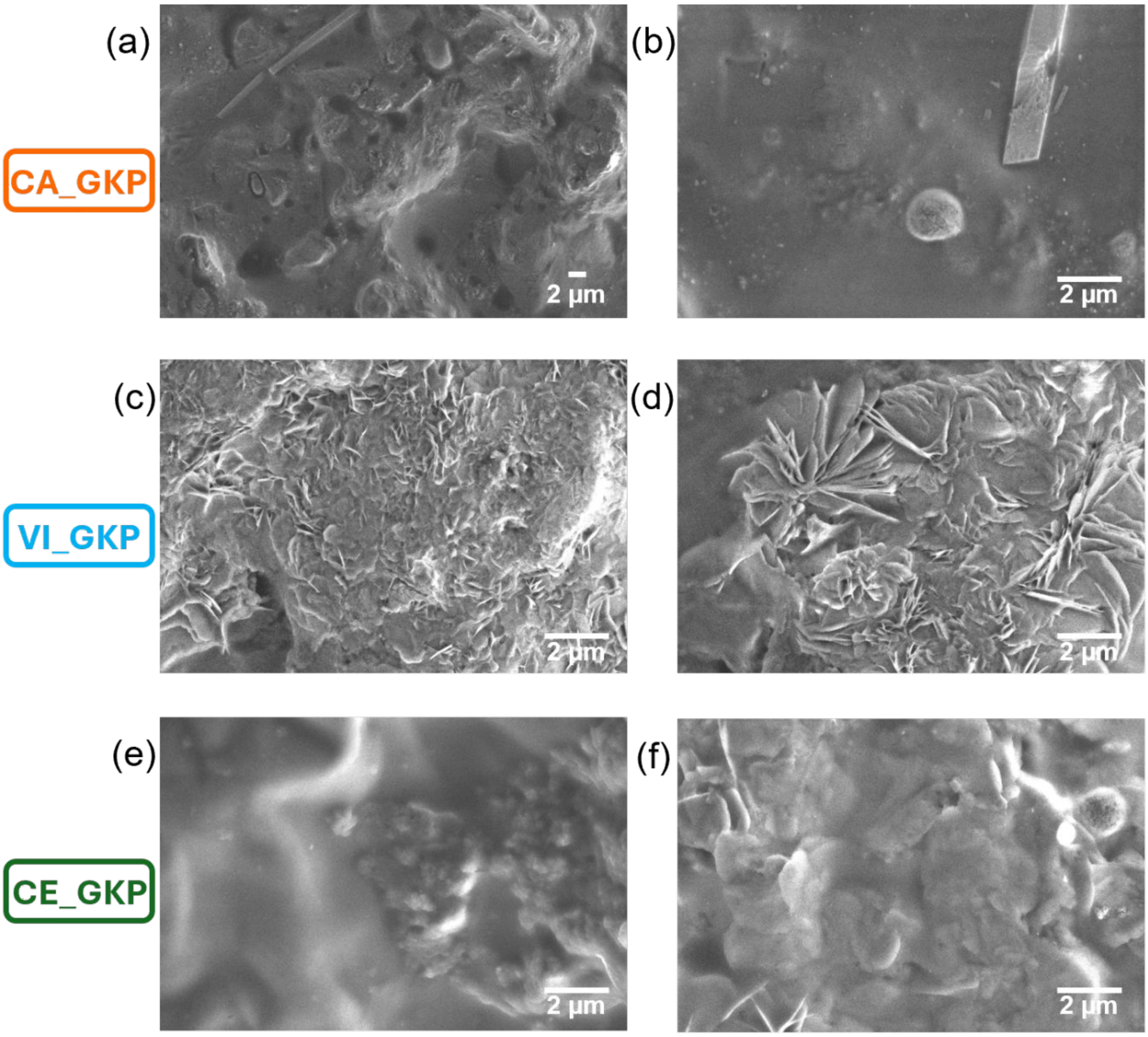

Figure S2. SEM images with 20.000 X magnification of (a),(b) CA_GKP, (c),(d) VI_GKP and (e),(f) VI_GKP films on both air-dried and silicon-dried sides respectively from left to right.

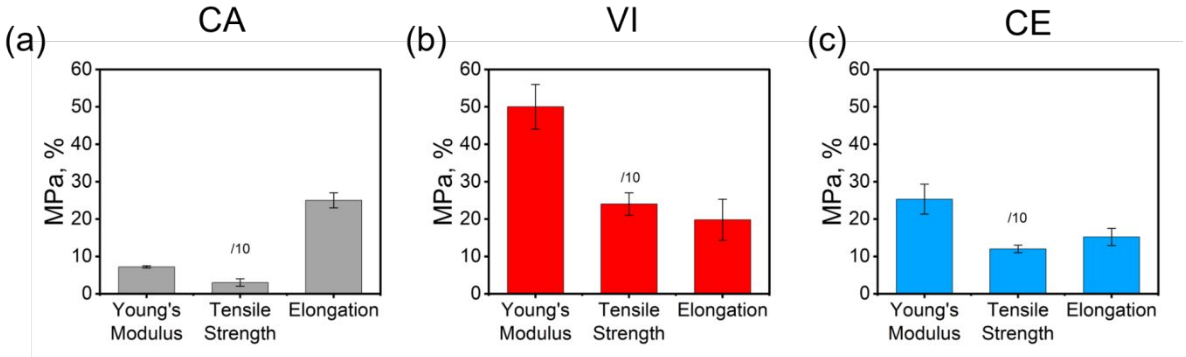

Figure S3. Mechanical properties of GKP films in the absence of $CaCl_2$. Mechanical properties of CA_GKP (a), VI_GKP (b), CE_GKP (c) films treated without $CaCl_2$ salt. Values are reported in Table S3.

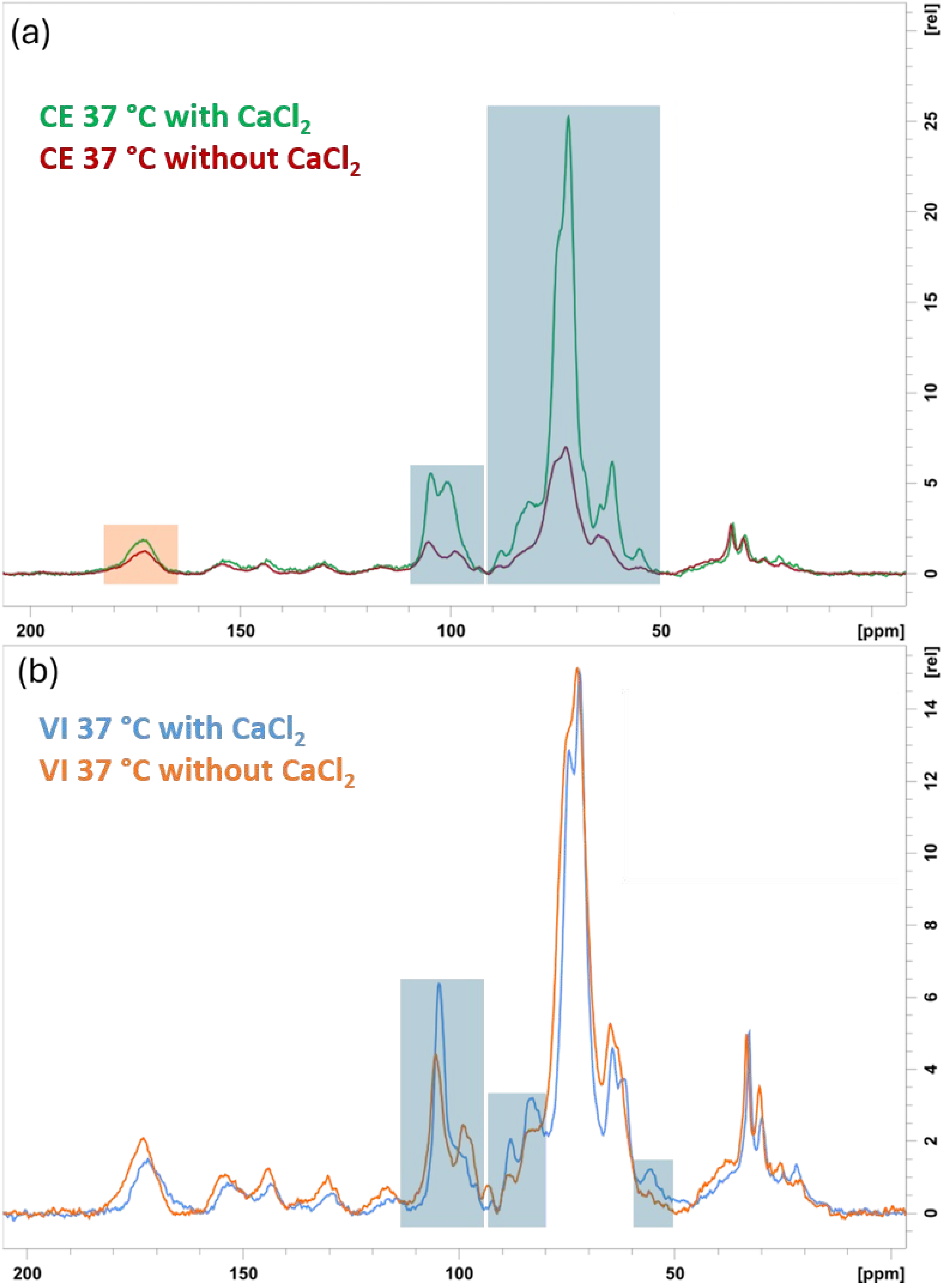

Figure S4. CPMAS ^{13}C-NMR spectra of GKP powder treated without $CaCl_2$. Regions that differ from the spectrum of GKP powder treated in presence of $CaCl_2$ are highlighted with colors corresponding to the different components (red for pectin, green for hemicellulose/cellulose).

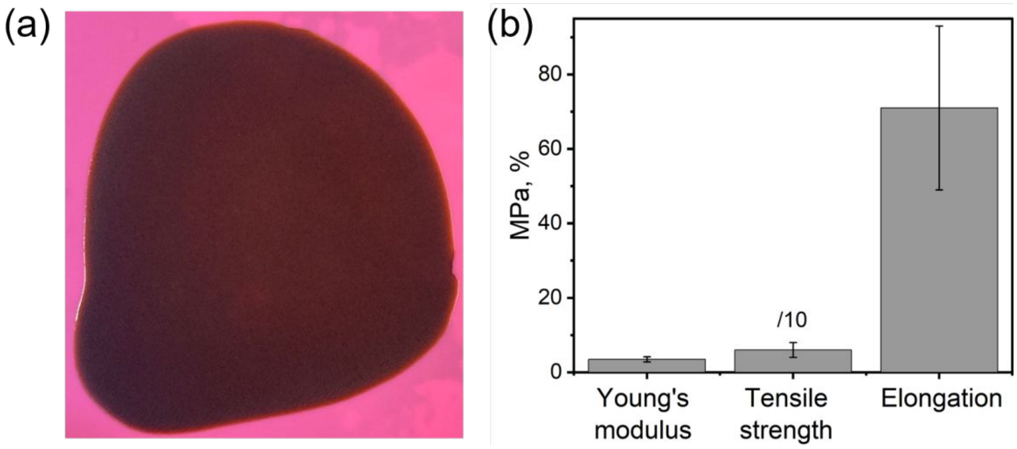

Figure S5. (a) Picture and (b) mechanical properties of film obtained with GKP treated with CA at 37 °C.

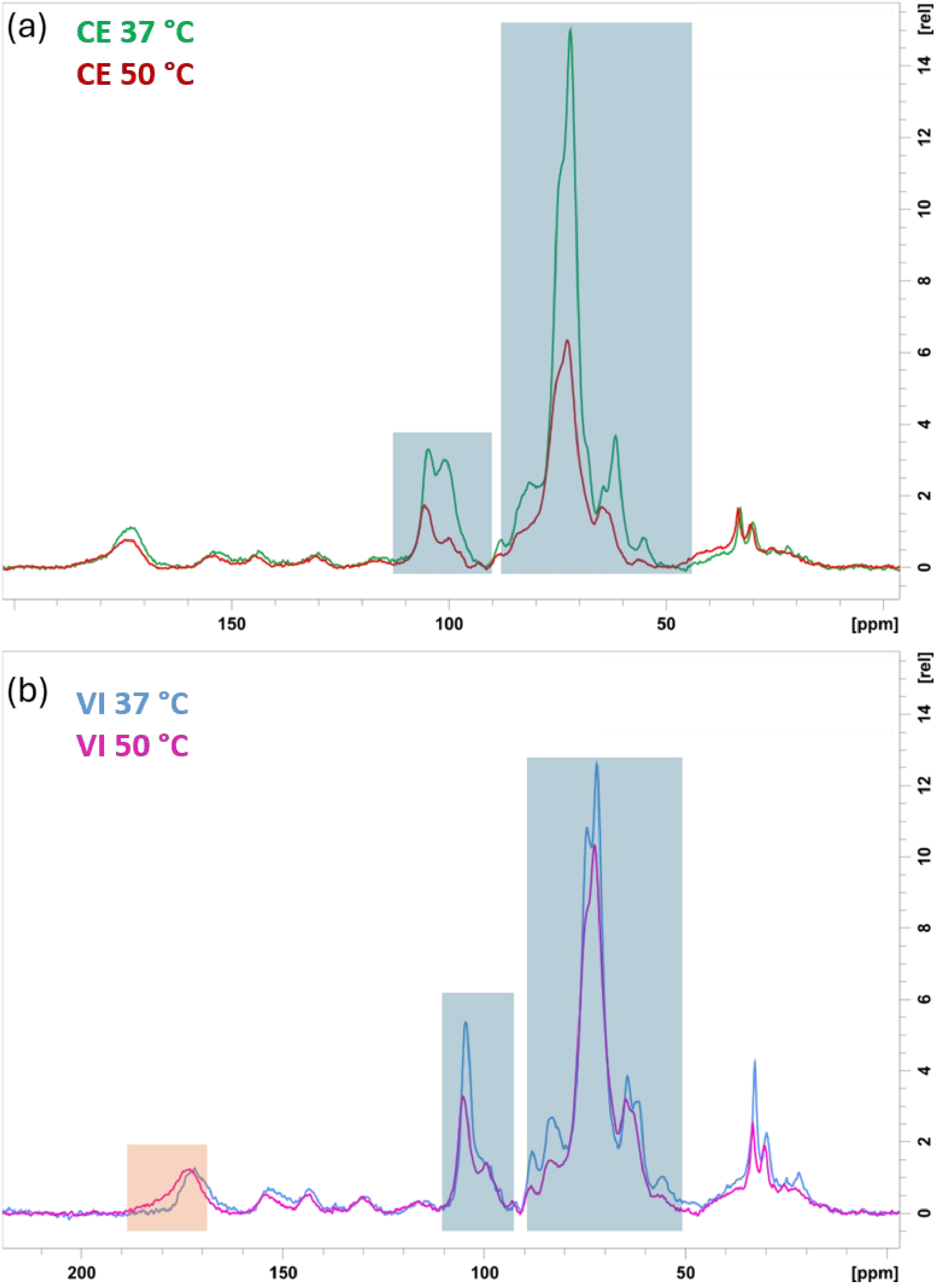

Figure S6. CPMAS ^{13}C-NMR spectra of GKP powder treated with (a) CE and (b) VI at 50 °C. Regions that differ from the spectrum of GKP powder treated at 37 °C are highlighted with colors corresponding to the different components.

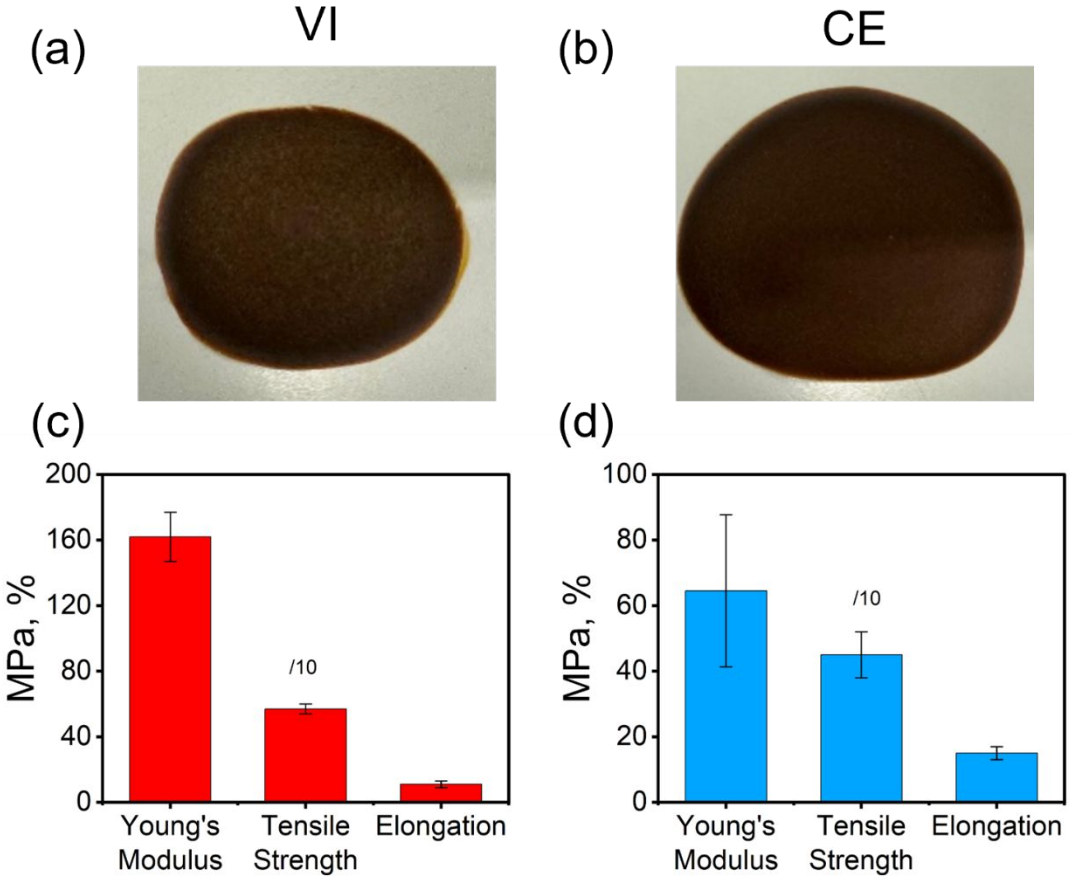

Figure S7. Properties of films obtained with GKP treated with VI and CE at 50 °C in the presence of $CaCl_2$. Photographs of VI_GKP (a) and CE_GKP (b) films and their relative mechanical properties. Values are reported in Table S1.
